# MC-GenomeKey: a multicloud system for the detection and annotation of genomic variants

**DOI:** 10.1186/s12859-016-1454-2

**Published:** 2017-01-20

**Authors:** Hatem Elshazly, Yassine Souilmi, Peter J. Tonellato, Dennis P. Wall, Mohamed Abouelhoda

**Affiliations:** 1 Center for Informatics Sciences, Nile University, Juhayna Square, Sheikh Zayed, Giza, Egypt; 2 Systems and Biomedical Engineering Department, Faculty of Engineering, Cairo University, Giza, Egypt; 3 Department of Biology, Mohamed Vth University in Rabat, 4 Ibn Battouta Avenue, BP: 1014RP, Rabat, Morocco; 4 Department of Biomedical Informatics, Harvard Medical School, 10 Shattuck Street, Boston, MA 02115 USA; 5 Department of Pathology, Brigham and Women’s Hospital, Harvard Medical School, Boston, MA 02215 USA; 6 Department of Pediatrics and Psychiatry (by courtesy), Division of Systems Medicine & Program in Biomedical Informatics, Stanford University, Stanford, CA 94305 USA

**Keywords:** Variant analysis, Cloud computing, Multicloud, Sequence analysis, Personalized medicine

## Abstract

**Background:**

Next Generation Genome sequencing techniques became affordable for massive sequencing efforts devoted to clinical characterization of human diseases. However, the cost of providing cloud-based data analysis of the mounting datasets remains a concerning bottleneck for providing cost-effective clinical services. To address this computational problem, it is important to optimize the variant analysis workflow and the used analysis tools to reduce the overall computational processing time, and concomitantly reduce the processing cost. Furthermore, it is important to capitalize on the use of the recent development in the cloud computing market, which have witnessed more providers competing in terms of products and prices.

**Results:**

In this paper, we present a new package called MC-GenomeKey (Multi-Cloud GenomeKey) that efficiently executes the variant analysis workflow for detecting and annotating mutations using cloud resources from different commercial cloud providers. Our package supports Amazon, Google, and Azure clouds, as well as, any other cloud platform based on OpenStack. Our package allows different scenarios of execution with different levels of sophistication, up to the one where a workflow can be executed using a cluster whose nodes come from different clouds. MC-GenomeKey also supports scenarios to exploit the spot instance model of Amazon in combination with the use of other cloud platforms to provide significant cost reduction. To the best of our knowledge, this is the first solution that optimizes the execution of the workflow using computational resources from different cloud providers.

**Conclusions:**

MC-GenomeKey provides an efficient multicloud based solution to detect and annotate mutations. The package can run in different commercial cloud platforms, which enables the user to seize the best offers. The package also provides a reliable means to make use of the low-cost spot instance model of Amazon, as it provides an efficient solution to the sudden termination of spot machines as a result of a sudden price increase. The package has a web-interface and it is available for free for academic use.

## Background

The revolutionary Next Generation Sequencing (NGS) technology has provided a cost effective, fast, and efficient means for large scale detection of variants (mutations) in the genome. In medicine, the value of NGS for variant detection is continuously increasing, not only in the research domains but also in the daily clinical practice, largely for diagnosis and prognostics and growingly for treatment as well. The computational workflow for variant analysis is a multistep process that includes spotting the variants with high accuracy, evaluating their effect, and reporting all pieces of knowledge related to them. For human samples, the requirements of quality, accuracy, speed, and integrated knowledge sources are higher, compared to samples from other organisms. Such sophistications render the pipeline more complex and more data intensive, and hence necessitate the availability of high performance computing resources and storage that can be allocated in a reliable and quick manner.

Cloud computing is another revolutionary technology that has changed the way computational resources are made available. Concepts like virtualization, on-demand allocation of machines, and key-value storage became familiar to all scientists from different domains. Commercial cloud computing service providers took the lead in introducing cloud computing services on a pay-as-you-go basis and with well-defined pricing schemes. Academic clouds use the same concepts, but they usually offer limited resources for free, usually based on the first-in-first-service principle. Amazon Web Services (AWS) [1] has pioneered the provisioning of cloud computing services. It then followed by other providers like Microsoft Azure [2] and Rackspace [3]. Very recently, Google (Google Compute Engine GCE) [4] has arrived at the scene and offered very competitive products compared to Amazon.

The use of cloud computing as a cost effective and scalable infrastructure for running the variant analysis workflow has been evaluated in different studies [5–10] and a number of ready-to-use systems have been developed. In Academia, there are Games [11], Simplex [12], Atlas2 [13], and StormSeq [14]. In industry, the cloud based versions are accessed through web-interface, usually on a subscription basis. Important examples coming from major NGS manufacturers include BaseSpace from Illumina [15] and IonReporter from LifeTech [16].

The above mentioned variant analysis systems run only in Amazon’s cloud. The use case scenario of using that cloud platform is composed of the following steps: the user instantiates virtual machines at their own cost using pre-configured machine images deposited in Amazon. The instantiated virtual machines include middleware packages that are then invoked to establish a computer cluster. Example middleware packages include StarCluster [17], Vappio [18], and elasticHPC [19]. Once the cluster is configured, the input data are either uploaded to the shared cluster storage from the local computer or read from the Amazon storage S3. Once the data transfer is complete, the execution of the analysis task(s) starts. When the computation is over, the results are made available for download or deposited in S3 Amazon storage. Finally, the virtual machines are terminated.

The latest IaaS (Infrastructure as a Service) products of Google and Azure in combination with large price reduction have changed the landscape of the cloud computing market. They attracted many users and resulted in a segmented market, which accordingly would profit the clinical grade processing of NGS data. Mid 2015, Google announced new pricing schemes that have driven cloud prices further down. Google introduced about 30% reduction in prices compared to Amazon for most of the on-demand virtual machine types. Furthermore, Google charges per minute (after the first 10 min), while Amazon still charges per hour. Up to this date (September 2016), the Amazon prices did not change, but one would expect so soon due to the severe competition.

To cope with the current market status and to satisfy clinical practice needs, it is important to provide new cloud-based bioinformatics packages supporting multiple clouds: First to serve different users registered in different cloud systems, and second to seize best sales offers. Furthermore, the new cloud systems should also be able to run in a hybrid mode using cloud resources from different providers (multi-cloud) for the same workflow to 1) optimize the performance, 2) reduce the cost, and 3) provide a sort of fault tolerance with smooth non-interruptible execution, by migrating failed tasks in one cloud to another. In other words, the end user would gain the following advantages:

- Run the workflow on the cloud platform of choice, without adhering to one provider.
- Make use of new virtual machine offers (new high performance machines) and seize best discounts.
- Have a kind of redundancy in case of failure of one cloud site. The response to failure can even take place in execution time and the workflow can migrate to another cloud site.
- Mix cloud resources and best offers from different cloud service providers, which can indeed help reducing the overall cost. (Like mixing the use of spot instance model of Amazon with the use of Google machines).

### Contribution

In this paper, we present MC-GenomeKey, a multi-cloud based package for variant detection and analysis. Our package enables the users to run the workflow either in Amazon, Google, Microsoft Azure, or on any platform supporting OpenStack interface or Amazon-like interface. We support two main multi-cloud use scenarios: First, the whole workflow can run entirely in one cloud environment. Second, parts of the entire workflow can run in one cloud site and other parts can run in another cloud. In the second scenario, it is possible to distribute whole tasks or even individual jobs of the same task to different cloud sites. The use of multiple cloud resources in MC-GenomeKey can be decided in the design time or in the run time in response to some events requiring re-distribution of tasks or jobs on different clouds. As we show by experiments, the availability of these scenarios will help the user reach the best performance at lowest cost.

In addition to its novel (multi) cloud features we introduce in this paper, MC-GenomeKey package has superior features compared to the currently existing solutions. In Table 1, we compare the features of MC-GenomeKey to that of STORMseq [14], Atlas2 [13], Simplex [12], and WEP [20], and the previous single-cloud version of GenomeKey [21]. Notably, MC-GenomeKey has advanced and robust parallelization technique that runs in a computer cluster. Furthermore, it has the feature of analyzing multiple samples in a batch mode, saving the total execution time and cost. It also includes a comprehensive annotation of variants based on the Annovar package [22]. Finally, the proposed solution is modular, where each tool can be easily replaced by a newer version or another one.

**Table 1:** Comparison of different systems for variant analysis

	STORMseq	Atlas2	Simplex	WEP	GenomeKey	MC-GenomeKey
Quality	-	-	fastx-toolkit	ngs-qc toolkit + fastqc	fastx-toolkit	fastx-toolkit
Mapping	BWA	-	BWA	BWA	BWA	BWA
Variant Calling	GATK	Logistic Regression Model	GATK	GATK	GATK	GATK
Annotation	VEP (variant effect predictor)	-	Annovar	Annovar	Annovar	Annovar
Deployment	AWS EC2	AWS EC2	AWS EC2	Web Service	AWS EC2	AWS, Google Cloud, Amazon, OpenStack based
Web Interface	Yes	Yes	No	Yes	Yes	Yes
Multiple samples in one run	No	No	No	No	Yes	Yes
Parallelization technique	split by chromosome	-	-	NA	split by chromosome + split by read group id	split by chromosome + split by read group id + more split by sub-group ID and sub-chromosomes
Workflow Engine	Python Scripts	-	JClusterService	Scripts	Cosmos	Cosmos
Modularity	No	-	No	No	Yes	Yes
Use of Heterogeneous^a^ cluster	No	No	No	NA	No	Yes
Failure-handling^+^ Mechanisms	No	No	No	No	No	Yes
Use of Spot Instances	No	No	No	NA	No	Yes

^a^Heterogeneous cluster means nodes of different virtual machine types and also from different clouds

^+^Failure handling means response to failure of compute nodes in cloud, as in the case of spot instances

Providing a package implementing these scenarios in a multi-cloud setting is not a straightforward task, because all of the aforementioned clouds are built with different architecture, usage scenarios, APIs, and business models. Furthermore, providing such scenario requires changes in the workflow execution model and in the cluster middleware to cope with this heterogeneity. All these challenges will be addressed in the implementation section of the paper.

## Implementation

### MC-GenomeKey basic features

MC-GenomeKey is a package implementing the variant detection and analysis workflow, based on the python-based Cosmos workflow engine [23]. Its basic features can be categorized into three groups: 1) Variant Analysis Workflow Specifications, 2) Workflow engine and parallelization schemes and 3) Cloud Support. The details of these features are as follows:

### Variant analysis workflow specifications

**Fig. 1:**
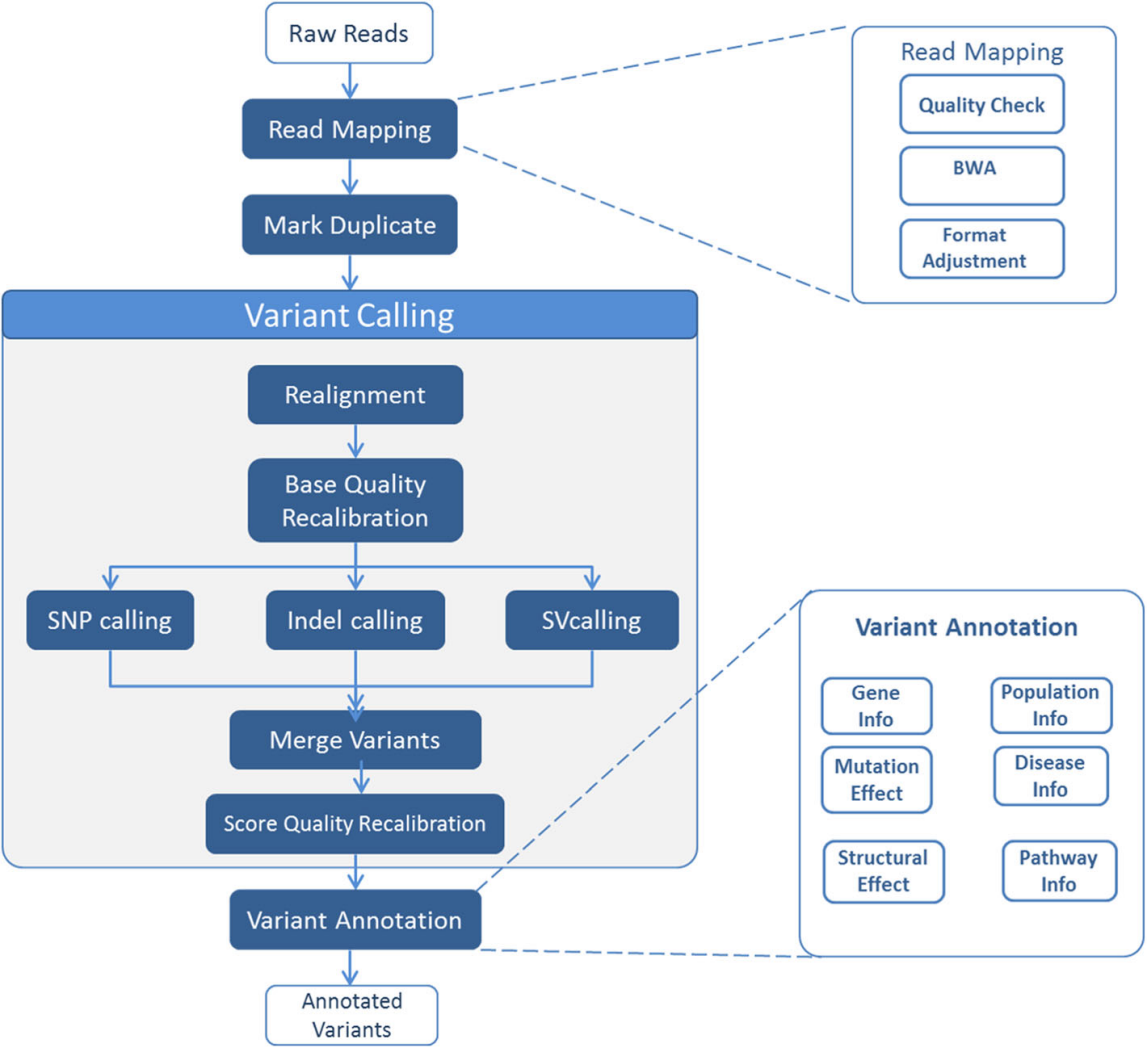
Variant Analysis Workflow. Variant Analysis Workflow. The figure shows the major phases and other major steps in each stage

Figure 1 shows the basic phases of the workflow for variant detection and annotation. Here is a description of these steps.

- ***Quality Check***: This is to verify the quality of the input read and trimming out the low quality terminal parts of the reads. The default program for this steps is the Fastx [24] toolkit.
- ***Read Alignment***: This is to map the reads to the reference human genome (hg19 ND GRCh38 are the default versions). The default alignment program is BWA [25].
- ***Variant Calling***: This is to analyse the read alignment file to determine the positions where the variants exist. The default program for this is the GATK variant caller [26]. The variant calling step itself is a pipeline including a number of operations: it includes realignment of reads around the variant, quality score recalibration, and generation of the variant file in a VCF format.
- ***Variant Annotation***: This is to annotate all the variants with all possible knowledge from different structural, functional, and population databases. The default system used for annotation is the Annovar package [22].

#### Usability

MC-GenomeKey is a user friendly package. The workflow can be invoked using a desktop client or from the package web-site. The parameters for each step can be set through a configuration file in case of the command line interface or through a web-form when using the web-interface.

**Fig. 2:**
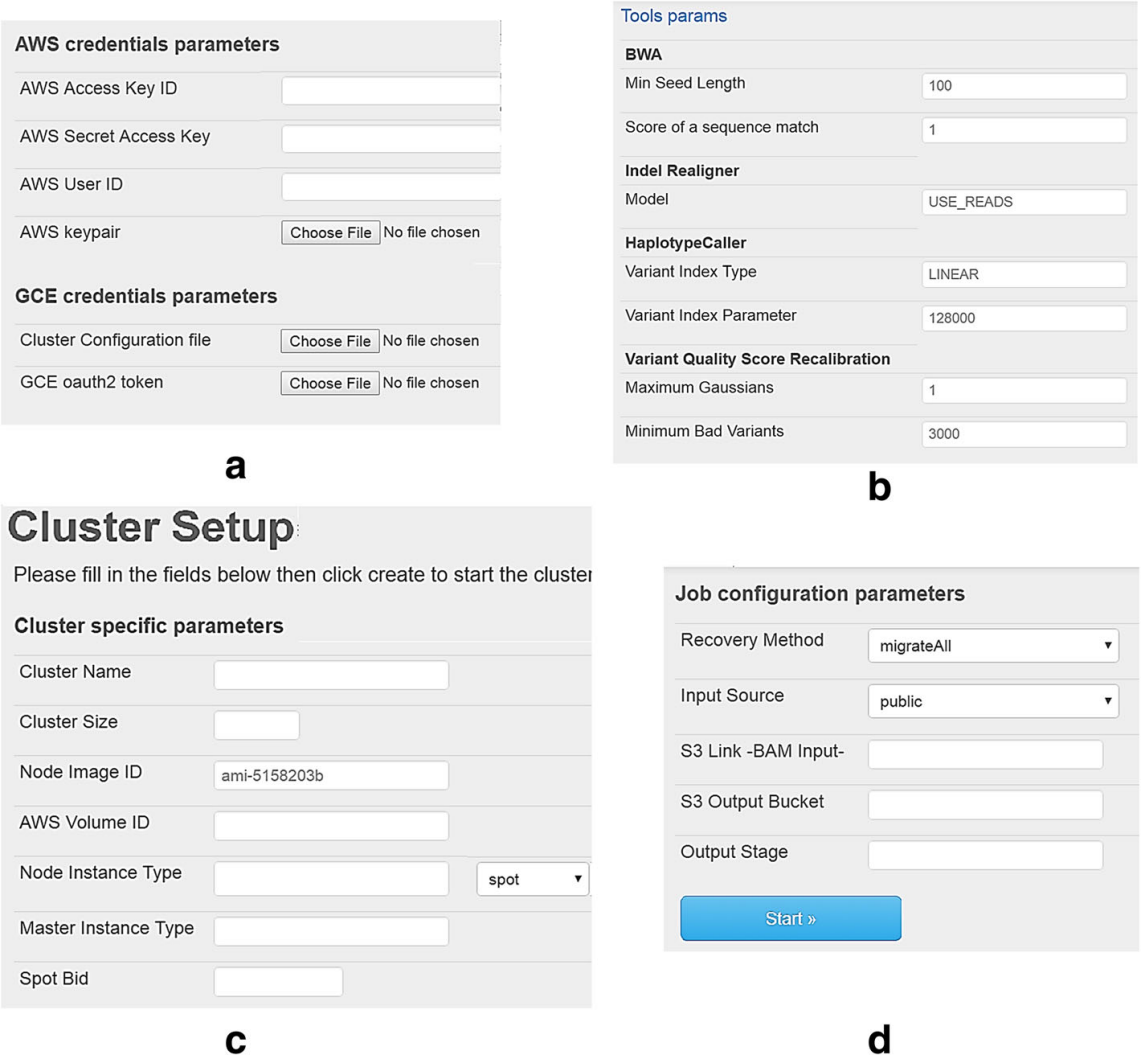
Screenshots of the MC-GenomeKey website. Screen shots of the web interface: **a** the user enters own credentials for Amazon and Google cloud. **b** The user sets workflow parameters, e.g., alignment and variant calling parameters. **c** The user defines the size of cluster, type of nodes, and use of spot instances or not. **d** The user sets the job configuration parameters, where the user can select the “recovery method” to respond to termination of spot instances. In this screen the input and output folders are defined

Setting up the cloud cluster is achieved via a simple interface, where the user defines the number and type of virtual machines. The use of different clouds requires the user’s credentials registered in each cloud site to run the system in the respective cloud. Therefore, the user provides own credentials for each cloud beforehand. For Amazon and Azure clouds, the user enters credentials in the form of certificates and private keys. For Google, the user provides an oauth2 token. To facilitate the generation of the token for Google, we provide a web-based form in the MC-GenomeKey website that forwards the user to Google to manage own cloud resources and authenticate the application. (We also provide the source code for this step so that the user can run it from own local server.) In case of using the MC-GenomeKey web-site, the user is recommended to disable all certificates and tokens after completion of computation. Apart from the security issues, all other technical details related to the setup of the cloud resources and parallel execution of the workflow is kept hidden from the user. Figure 2 shows some screen shots of the web-interface.

When the computer cluster starts in the cloud, an additional website is automatically generated in the master node of the cluster in order to monitor the cluster and to manage the nodes in the run time. There are also pages to monitor the execution of the analysis workflow. The access to these pages is explained in the package manual.

### Workflow engine and parallelization scheme

**Fig. 3:**
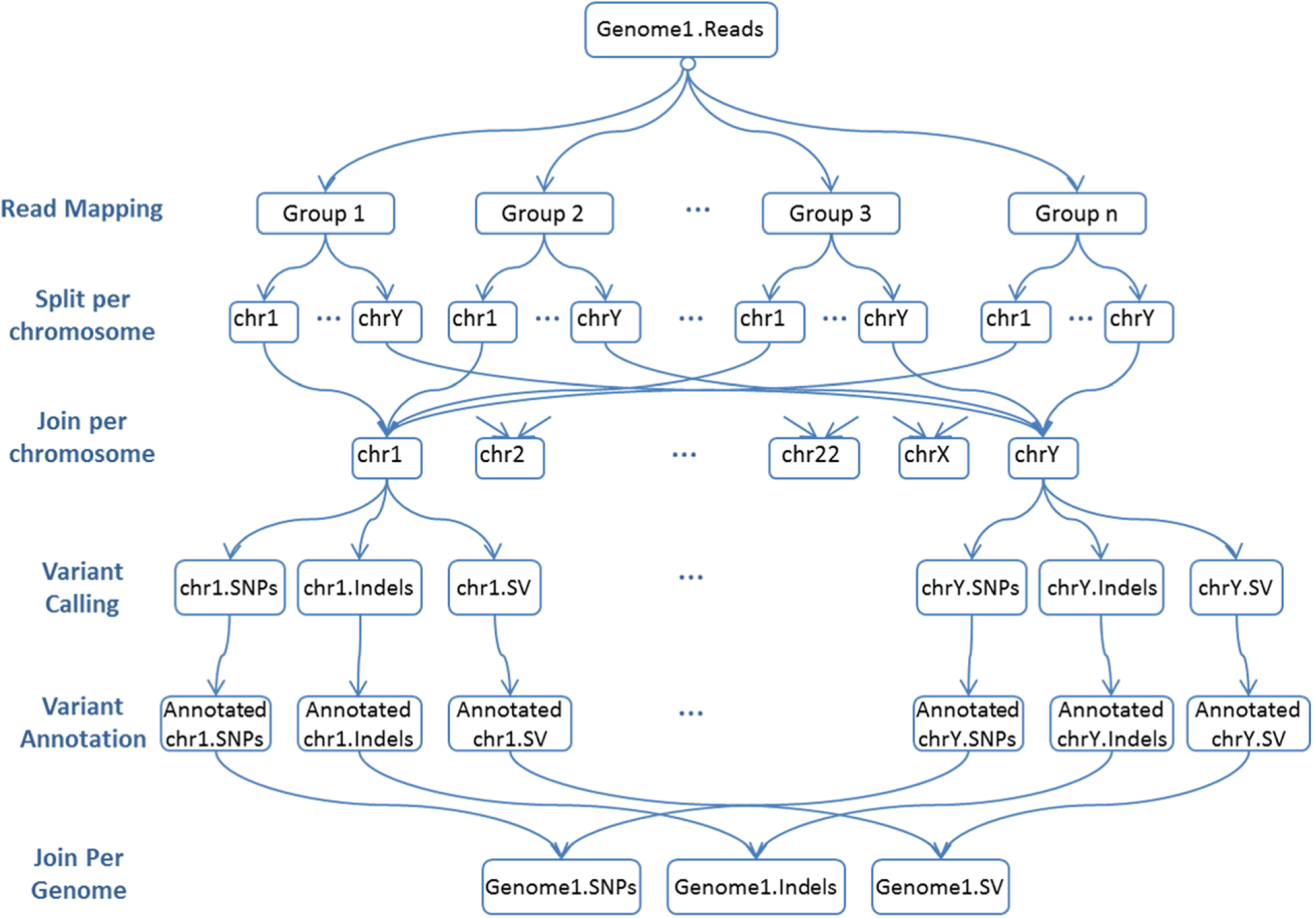
Data flow associated with the parallel execution of the MC-GenomeKey jobs. Data flow associated with the jobs of MC-GenomeKey in the form of DAG (Directed Acyclic Graph). A job is executed only if all the input became available

MC-GenomeKey is based on the python-based workflow engine Cosmos [27], which is optimized to run data analysis jobs in parallel over a computer cluster. Once the input NGS data is defined, the different steps of MC-GenomeKey are executed using the Cosmos engine as follows: MC-GenomeKey first creates a directed acyclic graph (DAG) of job dependencies, where the jobs are defined based on partitioning the input data (when executed). The DAG assures the correct execution of the workflow as it assures that a job is executed only when its input is made available from previous jobs. Figure 3 shows a simplified example DAG and the associated data flow for the variant detection and analysis workflow.

The parallelization scheme works as follows: The set of input reads is partitioned into blocks based on the read group IDs. Once the alignment is produced, the aligned reads are then partitioned by chromosomes. This way of partitioning was already implemented in the first version of GenomeKey [21], but the associated scalability is limited, because 1) the read group field is a characteristic of the Illumina sequencing platforms and might not be properly filled with other platforms, and 2) the maximum number of jobs for the read mapping step that can run in parallel cannot exceed the number of read groups, and the maximum number of jobs for the variant caller cannot exceed the number of chromosomes. To improve this situation, we have implemented a more flexible and advanced parallelization scheme in this version of MC-GenomeKey that works as follows: The input BAM is processed to increase the number of read groups, by creating sub-read groups associated with a parent read group, which increases the number of jobs that can run in parallel for the read mapping step. In other words, the read groups are re-written or properly added to the BAM file. The same idea is also adopted for processing the reads from the same chromosome in parallel, where we define sub-chromosomes corresponding to large segments of the chromosome. The splitting by sub-chromosomes is optional in the package and is allowed only for large segments (default 50 MB) so that the statistics for the variant calling is not affected.

The data flow among the tasks/jobs of the workflow is achieved by using files and each task/job recognizes its input by certain file extensions associated with the respective job ID. These intermediate files are automatically managed by the engine. The parallelization of job execution over the cluster nodes is managed by the DRMAA package [28], which encapsulates different job schedulers (CGE, Condor, etc) and efficiently deals with job submissions, monitoring, and errors using single common interface. To make the data available for all cluster nodes, a shared storage in the form of EBS volume is created and mounted to the master node. The input is moved to this shared storage from the user’s computer or from the user’s S3 account before computation. The intermediate data is kept in the shared storage. This shared storage remains alive even if all spot instances get terminated; this is because it is attached to the on-demand master node.

### Cloud features

MC-GenomeKey supports different scenarios for using the cloud computing resources. These include the following:

- Individual Cloud: In this scenario, the user selects the cloud platform to be used. Unlike other cloud-based variant analysis systems, MC-GenomeKey can support Amazon, Google, and Microsoft Azure. The user is prompt to enter the credentials of the cloud of choice, the path to the input data, set the parameters in the configuration file, and starts the workflow execution. MC-GenomeKey then launches the process of creating the computer cluster in the cloud using the deposited machine images and returns process ID to the user. The user does not need to be persistently connected to the internet. One can disconnect and use the process ID anytime to monitor the status of execution using another command line or using the web-interface. Once the process is over, the results are deposited to persistent cloud storage or are downloaded to the user local machine. The cloud computer cluster is finally terminated and a report is generated.
- Multi-Cloud: There are different forms to use multi-cloud either topological or temporal. Topological forms specify where the tasks and jobs are executed. Considering the variant analysis workflow, we identify three forms:
  - ○Replication: the same workflow runs simultaneously on different clouds on different datasets to increase the throughput, or to overcome some limitations at one site.
  - ○Task distribution: Different tasks are executed in different clouds. For example, the task of read mapping can run in Amazon and the remaining workflow can run in Google. In this case, different job queues can be used, but there should be a master task queue to manage the tasks themselves. Note that the task queue is handled by the workflow engine itself, while the jobs are managed by the job scheduler engine wrapped by the RDMAA in each cluster.
  - ○Job distribution: Certain jobs within the same task run in one cloud and other jobs run in another cloud. For example, in case of NGS reads of different lengths, one can run the alignment of longer reads in one cloud and shorter reads in another cloud. The distribution of jobs on different clouds requires that one master job queue manages the jobs over the different cloud sites.

The creation of a computer cluster with separate job queues or shared queue is supported by the multicloud version of the elastiHPC package [29].

Temporal forms address the time when multiple cloud resources are allocated. For the variant analysis workflow, assume, for example, that the user decides that task A runs in cluster C_1_ in one cloud and the subsequent task B runs in cluster C_2_ in another cloud. To save cost, C_2_ is created only when task A is over or when it starts to produce output. Also some tasks can start in cloud C_2_ in case of failure of some jobs in cloud C_1_. In other words, when jobs have to be migrated to finish the computation. In MC-GenomeKey, we support that each phase can run in different cloud and we support the migration of tasks in case of failure of some nodes to other nodes in another cloud.

Of course, the use of multicloud scenarios will involve the transfer of intermediate results using the Internet, which is a bottleneck for adopting these scenarios. Nevertheless, in the following paragraphs, we will present one use case, where the use of multi-cloud can effectively contribute to a more cost effective and reliable usage of the cloud.

### Multicloud and spot instances

The use of multicloud scenario can be of great benefit when using spot instances of Amazon. The spot instance model of Amazon is about the use of computing resources at lower prices, when Amazon environment is not fully loaded. How far prices get reduced depends on the load and this continuously changes over time. The user who wishes to use spot instances has to bid for a price. If the instance price becomes lower than the bid price, then instances are initiated. If the instance price becomes higher than the initial bid price, then the running instances are terminated without notice. In general, the price of a spot instance is much less than that of the equivalent on-demand instance, which is a very attractive feature. Table 2 shows different machine types with their on-demand and spot prices in Amazon. It also includes the prices of equivalent on-demand machines in Google. It can be directly observed that the spot instances are the best to use to save cost. However, the major risk with the spot instance model is that the machines can terminate before the computation is over, and this risk is not rare to happen. Table 3 shows different statistics about the lifetime of different spot instances against different bid prices. This table was computed based on history information of the Amazon sport instances; the AWS command describe-spot-price-history (AWS documentation) was used to retrieve the price information for a period of three months. It can be observed that the average lifetime increases with the larger bid price. It can also be observed that the higher the machine specification the shorter the lifetime. This means that the average lifetime can be enough to finish short-time workflows, but there is a high probability to lose spot instances for long-time workflows.

**Table 2:** On demand prices for Amazon and Google instances

	Amazon					Google		
Instance type	CPUs	Mem	Price	Spot Price	Instance Type	CPUs	Mem	Price
m3.2xlarge	8	30	$0.53	$0.07	n1-standard-8	8	30	$0.28
c3.8xlarge	32	60	$1.68	$0.25	n1-standard-16	16	60	$0.56
c4.xlarge	36	60	$1.76	$0.27	n1-highcpu-32	32	28.8	$1.216
r3.8xlarge	32	104	$2.66	$0.3	n1-standard-32	32	120	$1.12

Spot instance prices for Amazon are the minimum prices observed such that the instance was available for at least one hour (prices are computed for three months period from October until December 2015)

**Table 3:** Life Time of different machines (in minutes) against different bid prices

c3-8xlarge				
Bid	Average	Minimum	Maximum	Median
$0.2	0	0	0	0
$0.3	89.22	1	2401	8
$0.4	201.17	1	5168	14
$0.5	442.18	1	15907	15.5
$0.6	3311.57	1	51244	29.5
$1.00	17272.6	1	65193	12
c4-8xlarge				
Bid	Average	Minimum	Maximum	Median
$0.2	0	0	0	0
$0.3	185.23	1	3921	11
$0.4	386.27	1	15880	14
$0.5	825.84	1	30899	10
$0.6	1296.8	1	30899	15
$1.0	5359.12	2	32511	26.5
r3-8xlarge				
Bid	Average	Minimum	Maximum	Median
$0.2	0	0	0	0
$0.3	64.58	1	713	16
$0.4	139.26	1	4811	14.5
$0.5	175.65	1	8318	18.5
$0.6	229.79	1	8344	25
$1.0	422.08	1	14441	23.5

Prices are computed for three months period from October until December 2015. Instances of type m3-2xlarge were available all the period with a bid price of 0.2$

To overcome the dilemma associated with the spot instances, one can use a multicloud computation environment which is a mix between Amazon and Google instances to assure termination of computation with reduced cost and minimal overhead. The cost reduction stems from the spot instance model and the fact that Google is cheaper than Amazon and it charges the user per minute and not per hour.

MC-GenomeKey offers three alternative solutions to the problem of sudden termination of spot instances: 1) Wait-and-Rebid, 2) Continue with on-demand instances in the same cloud, and 3) Migrate execution of failed jobs to another cloud. Each of these solutions will be discussed in the following paragraphs. The last two scenarios are based on the multi-cloud to achieve the best performance and cost.

**Fig. 4:**
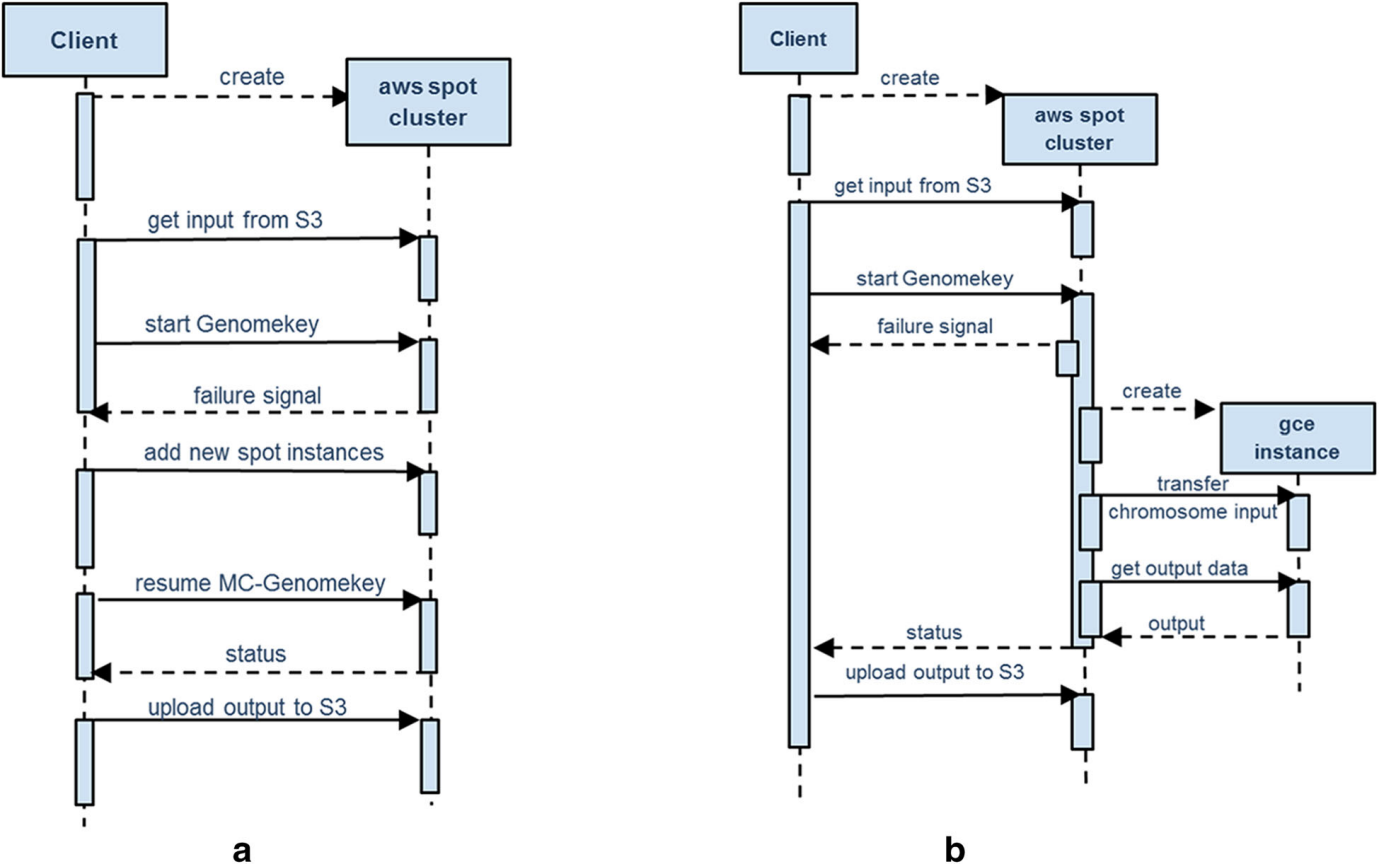
Scenarios for handling sudden termination of spot instances. Sequence diagrams showing scenario 2 (**a**) where the computation continues in the same Amazon (AWS) cloud and Scenario 3 (**b**) where computation filed jobs in terminated spot instances are migrated to Google (GCE) cloud
- **Scenario 2, Continue in the same cloud with on-demand nodes:** In this scenario, the failed spot instances are replaced with on-demand instances in the same cloud. The job scheduler is set-up to remove the failed ones and add the new ones.
- **Scenario 3, Migrating failed jobs to Google Cloud:** As depicted in Fig. 4b, MC-GenomeKey will replace terminated spot worker nodes in Amazon with other ones in Google. The failed jobs on the terminated spot nodes will be moved to the new nodes and re-executed there. This scenario includes the case where all the cluster nodes are spot instances that get terminated and the execution of the variant analysis pipeline switches completely to Google.

For ease of presentation, we assume that the master node of the main computer cluster in AWS is an on-demand node and other worker nodes are spot instances. The on-demand node keeps the workflow running, even if all spot instances are terminated and it supports the scenarios mentioned above to restore the computation power. This cluster of heterogeneous compute nodes can be readily created with the elasticHPC package [19, 29] without extra effort. In the following we summarize how the four scenarios will work:

- **Scenario 1, Wait and Rebid:** As depicted in the sequence diagram of Fig. 4a, if the price of any or all of the spot instances exceeds the bid price (out-bided), MC-GenomeKey will remove the failed instances from the cluster configuration. The user then uses the cloud-client software to make a new bid for the spot instances. When the new spot instance starts before the completion of the workflow, they are added to the currently running nodes and join the execution plan. For this scenario, MC-GenomeKey is modified to keep the intermediate data in the EBS volume (virtual disk) of the master node.

To optimize the performance and reduce the effect of data transfer over the internet, we do not transfer the whole data from the beginning, but only those needed to complete the tasks to run on Google. For example, if the failure occurs in the annotation step, then only the respective VCF files are sent to Google. Furthermore, we dispatch also the subsequent jobs following the failed ones in the DAG. This will prevent moving the data back and forth between the Amazon and Google instances; i.e., the data needed for computation is transferred only once and the workflow continues to the end. (This works correctly because of the DAG nature of the workflow, where each task needs data only from previous step.) When the workflow finishes successfully, the output of the Google instances will be transferred to the output directory in the AWS cluster on the shared storage and the whole directory will be compressed and uploaded to AWS S3. The default setting is that the intermediate data needed for computation in Google is moved from Amazon and it is deleted automatically when the google machines are terminated. In addition, we give the user an option to keep a copy of the data transferred to Google in Amazon and send the intermediate data generated by Google back to Amazon with the output.

### Extra implementation details

MC-GenomeKey is based on a client-server architecture. The user installs a client module at own desktop to control the analysis process. The client module uses the cloud provider APIs to manage the cloud resources. The client also controls the created instances, configures them, and starts the analysis workflow and follows its progress.

Specifying the job parameters, such as the workflow name, input and output path on S3 and output directory to upload to S3, and the response to spot instance failure (if used), are achieved through a workflow configuration file.

The multi-cloud version of elasticHPC is used to create and manage instances of the computer cluster in the selected cloud. Another configuration file is required for elasticHPC to specify the cloud resources, this file includes a number of nodes, machine types, storage, and security. The elasticHPC website includes detailed explanation of the configuration file. For each cloud environment, we have created a virtual machine image including all necessary settings and tools to the run the MC-GenomeKey on each cloud. The master and worker nodes of the cluster are created from the respective image. The master node also works as a server that responses to requests from the client and responses to any failure in the cluster nodes.

### Implementing the spot instance scenarios

Once the cluster nodes are setup in Amazon, a control program in the master node automatically starts to monitor the state of the running spot instances. This program reports node status to the user and initiates the recovery procedure as specified by the user in the job configuration file.

For the 3rd scenario, we updated COSMOS task database with a field to associate the task with the worker node (to identify failed tasks) and with the chromosome being processed on the GCE worker node (to merge results again). Also a specific queue is created to dispatch tasks to GCE instances in case of spot instance failure, this procedure is depicted in Fig. 4.

## Results and Discussion

### Experiment1: performance in different clouds

We measured the performance of MC-GenomeKey on AWS, Google, and Azure using the following two datasets:

- A whole genome dataset of 113 GB from [23]. The NGS reads come from Illumina NGS machines and cover all genomic regions with an average depth of about 30X.
- An exome sequence dataset (~9.2 GB), also from [23]. The NGS reads come also from an Illumina NGS machine and cover only the exons of the genome. Exome sequencing is important in many clinical applications, where clinicians search for disease-causing variants in the coding regions.

In AWS cloud, we used a master node of type m3.medium (0.067$/h) and four r3.8xlarge instances; each has 32 cores and 104 GB RAM and costs 2.66$/h. In Google, we used master node of type n1-standard-1 (0.035$/h) and four n1-standard-32 instances; each has 32 cores and 120 GB RAM and costs 1.6$/h. It is worth noting that GCE instances are charged per minute (after first 10 min). In Azure, we used master node of A2 instance family ($0.154/h) and four D14 v2 instances; each has 16 cores and 112 GB RAM and costs 2.428$/h.

In the established computer clusters, the master node was not an execution node, but it is a control node responsible for 1) the initiation and orchestration of the workflow and related data flow, 2) monitoring its status, and 3) carrying out the migration process from AWS to Google if needed. This configuration makes it possible to select a machine of lower specifications for the master node, which dramatically reduces the overall cost of the cluster in case of a migration scenario as will be explained below.

Table 4 shows the results of running the whole variant analysis workflow for the exome and whole genome datasets using a cluster of 4 nodes in different cloud environments. From the table we can observe that the performance on Amazon and Google is almost the same, and better than that of Azure. We also observe that the read mapping and variant calling are the most time consuming steps. The reason of cost reduction by Google compared to Amazon is the reduced machine price. Although Azure instances are cheaper than the equivalent ones in Amazon, the low performance raised the cost of using Azure to be higher than that of Amazon.

**Table 4:** Running times of the variant analysis workflow in different clouds

Exome (9.2 GB)			
	Amazon	Google	Azure
Alignment (BWA)	0:12:20	0:18:40	00:26:00
IndelRealigner	0:14:39	0:20:10	00:28:00
MarkDuplicates	0:15:29	0:23:06	00:35:00
BQSR	0:28:06	0:34:15	00:55:00
HaplotypeCaller	1:08:46	0:58:40	01:28:00
GenotypeGVCFs	0:14:23	0:12:40	00:17:00
VQSR	0:10:07	0:10:14	00:12:00
Merge VCF	0:05:07	0:04:55	00:10:00
Convert VCF to Annovar	0:00:13	0:00:10	00:00:15
Annotate	0:05:16	0:05:36	00:09:00
Merge Annotation	0:03:06	0:04:01	00:06:00
Total	1:51:33 ($21.414)	2:06:28 ($13.94)	3:12:00 ($31.24)
Whole Genome (113 GB)			
	Amazon	Google	Azure
Alignment (BWA)	8:03:53	8:10:13	11:18:20
IndelRealigner	3:04:03	3:09:34	04:15:58
MarkDuplicates	3:15:04	3:22:41	04:39:26
BQSR	4:11:14	4:17:23	06:35:44
HaplotypeCaller	9:05:43	9:15:49	14:29:22
GenotypeGVCFs	1:45:01	1:46:44	02:2:14
VQSR	1:33:29	1:33:36	02:05:13
Merge VCF	0:05:55	0:06:05	0:15:08
Convert VCF to Annovar	0:06:14	0:06:17	0:15:29
Annotate	0:15:01	0:15:21	0:24:39
Merge Annotation	0:10:06	0:11:01	0:23:07
Total	31:39:43 ($342.62)	32:13:06 ($208.07)	46:44:40 ($462)

Running times (hours: minutes: seconds) of the variant analysis workflow in different clouds using a cluster of 4 nodes.  We give the time of different steps. The total time and cost (in USD) are in the rows titled “Total”. The best running times and options are underlined

### Scalability using more nodes for the whole genome dataset

Table 5 shows the running times of the workflow using different cluster sizes running in Google Cloud. (The numbers in Amazon are analogous but not shown for ease of presentation.). We assured the correctness of the results, by comparing the resulting VCF files and verifying identical output. From the table, we observe good scalability with the increasing number of nodes.

**Table 5:** Total running times for running MC-GenomeKey on the whole genome dataset using different clusters of increasing node number

	Nodes			
	4	8	16	32
Alignment (BWA)	8:03:53	4:45:51	2:55:34	1:30:22
IndelRealigner	3:04:03	1:25:44	0:50:51	0:28:44
MarkDuplicates	3:15:04	1:35:01	0:48:15	0:33:10
BQSR	4:11:14	2:55:16	1:35:02	0:18:41
HaplotypeCaller	9:05:43	6:04:45	4:18:30	2:15:01
GenotypeGVCFs	1:45:01	1:01:10	0:37:14	0:15:09
VQSR	1:33:29	0:55:45	0:30:56	0:11:30
Merge VCF	0:05:55	0:08:39	0:11:20	0:13:15
Convert VCF to Annovar	0:06:14	0:07:11	0:06:59	0:07:01
Annotate	0:15:01	0:14:55	0:15:10	0:14:32
Merge Annotation	0:04:06	0:03:49	0:04:01	0:04:05
Total time	31:29:43	19:18:06	12:13:52	6:11:30

### Experiment 2: the spot model and live job migration

If the above workflow runs on Amazon using spot instances, it would cost much less than that of Google. However, the problem is that there is no guarantee that the machines are not terminated before the computation is completed. In this section, we provide experiments to measure Scenario 3 in case *all* and *some* worker spot nodes fail.

In case all spot instances fail, all tasks/jobs assigned to spot instances are migrated to Google cloud. Table 6 shows the times and costs when the machines are terminated at different time points while the workflow is executed, using different bid prices. We simulated the termination of spot instances at different time points of the workflow, by sending termination signals to the worker nodes. The created cluster automatically detects any termination of the nodes and executes the migration process. In case, some spot instances fail, same number of machines are created in Google and the tasks/jobs assigned to those nodes run there. Table 7 shows the time and costs when machines are terminated at different time points while the workflow executed, using different bid prices. For this table, we assume that half the spot instances fail.

**Table 6:** The cost of using spot instances for Case 1, where all spot instanced get terminated

	Bid = $0.2 (1 min)	Bid = $0.3 (65 min)	Bid = $0.35 (100 min)	Bid = $0.4 (140 min)	Bid = $0.5 (176 min)	Bid = $0.6 (230 min)	Bid = $1 (422 min)	Average data transfer time (cost)	Total time
Exome									
No Failure	$1.734	$2.534	**$2.934**	**$3.334**	**$4.134**	**$4.934**	**$8.134**	0	**1:51:33**
Failure Step 1 (Mapping)	**15.827**	16.227	16.45	16.627	17.027	17.427	19.027	12 min (0.96$)	02:08:23
Failure Step 2 (Variant Calling)	11.471	**11.871**	$12.1	12.271	12.671	10.937	12.537	11 min (0.87$)	02:06:10
Failure Step 3 (Annotation)	7.402	7.802	$8.1	8.202	8.602	9.002	10.602	6 s (0.00036$)	01:55:48
Whole Genome (4 Nodes)									
No Failure	$27.744	$40.544	$46.9	$53.344	$66.144	$78.944	$130.144	0	**31:39:43**
Failure Step 1 (Mapping)	**$229.87**	**$231.47**	**$232.28**	**$233.07**	**$234.67**	**$236.27**	**$242.67**	230 min (18.4$)	35:08:46
Failure Step 2 (VC)	$127.84	$135.84	$139.85	$143.85	$151.84	$159.84	$191.84	100 min (7.9$)	32:47:17
Failure Step 3 (Annotation)	$55.25	$84.59	$89.79	$94.99	$105.39	$115.79	$157.39	12 s (0.00432$)	32:14:01
Whole Genome (8 Nodes)									
No Failure	$32.23	$47.70	$55.42	$63.16	$78.63	$94.10	$155.96	0	**19:18:06**
Failure Step 1 (Mapping)	$243.75	**$245.35**	**$246.15**	**$246.95**	**$248.55**	$250.15	$256.55	230 min (18.4$)	23:10:1
Failure Step 2 (Variant Calling)	$123.05	$133.45	$138.65	$143.85	$154.25	**$164.65**	**$206.25**	100 min (7.9$)	20:52:08
Failure Step 3 (Annotation)	$54.99	$68.59	$75.39	$82.19	$95.79	$109.39	$163.79	12 s (0.00432$)	19:20:01
Whole Genome (32 nodes)									
No Failure	$39.88	$59.61	$69.50	$79.35	$99.08	$118.81	$197.75	0	**6:11:30**
Failure Step 1 (Mapping)	$291.75	**$294.95**	**$296.55**	$298.15	$301.35	$304.55	$317.35	230 min (18.4$)	10:45:12
Failure Step 2 (VC)	$172.65	$185.45	$191.85	**$198.25**	**$211.05**	$223.85	$275.05	100 min (7.9$)	7:56:03
Failure Step 3 (Annotation)	$85.39	$101.39	$109.39	$117.39	$133.39	**$149.39**	**$213.39**	12 s (0.00432$)	6:20:10

The cost of using spot instances with different bid prices and failure time points given for Case 1 where all spot instanced get terminated for the Exome and the Whole Genome datasets. GCE cluster setup time is nearly 7 min. For every bid we provided the average lifetime of cluster in brackets. Costs in bold are the most likely ones with the respective bid price and its most likely time of failure. The best expected costs for a given experiment are underlined. The best expected cost for exome is 3.334 using bid of 0.4 (underlined) and for whole genomes comes is 149.39 (underlined) with 32 nodes and bid price of 0.6

**Table 7:** The cost of using spot instances for Case 2, where some spot instances get terminated

	Bid = $0.2 (1 min)	Bid = $0.3 (62 min)	Bid = $0.35 (100 min)	Bid = $0.4 (135 min)	Bid = $0.5 (172 min)	Bid = $0.6 (224 min)	Bid = $1 (419 min)	Average data transfer time (cost)	Total time
Exome									
No Failure	$1.734	$2.534	$2.934	**$3.334**	**$4.134**	**$4.934**	**$8.134**	0	**1:51:33**
Failure Step 1 (Mapping)	$8.78	**$9.38**	$9.51	$9.98	$10.58	$11.18	$13.58	7 min (0.6$)	02:08:23
Failure Step 2 (Variant Calling)	**$9.13**	$9.73	$9.91	$10.33	$10.93	$11.53	$13.93	6 min (0.47$)	02:06:10
Failure Step 3 (Annotation)	$4.06	$4.66	**$4.81**	$5.26	$5.86	$6.46	$8.86	2 s (0.05$)	01:55:48
Whole Genome (4 Nodes)									
No Failure	$27.744	$40.544	$46.9	$53.344	$66.144	$78.944	$130.144	0	**31:39:43**
Failure Step 1 (Mapping)	$114.43	**$121.63**	**$125.23**	$128.83	$136.03	$143.23	$172.03	123 min (4.87$)	33:08:46
Failure Step 2 (VC)	$64.52	$74.92	$80.12	**$85.32**	**$95.72**	**$106.12**	$147.72	min (0.96$)	33:47:17
Failure Step 3 (Annotation)	$45.32	$56.92	$62.72	$68.52	$80.12	$91.72	**$138.12**	30 s (0.6)	32:14:01
Whole Genome (8 Nodes)									
No Failure	$32.23	$47.70	$55.42	$63.16	$78.63	$94.10	$155.96	0	**19:18:06**
Failure Step 1 (Mapping)	$137.64	**$146.04**	**$150.24**	$154.44	$162.84	$171.24	$204.84	123 min (4.87$)	21:30:1
Failure Step 2 (VC)	$77.33	$90.13	$96.53	**$102.93**	**$115.73**	**$128.53**	$179.73	101 min (0.96$)	21:12:08
Failure Step 3 (Annotation)	$48.26	$62.66	$69.86	$77.06	$91.46	$105.86	**$163.46**	30 s (0.585$)	19:55:01
Whole Genome (32 nodes)									
No Failure	$39.88	$59.61	$69.50	$79.35	$99.08	$118.81	$197.75	0	**6:11:30**
Failure Step 1 (Mapping)	$165.64	**$176.84**	**$182.44**	$188.04	$199.24	$210.44	$255.24	123 min (4.87$)	8:15:12
Failure Step 2 (VC)	$93.33	$109.33	$117.33	**$125.33**	**$141.33**	**$157.33**	$221.33	101 min (0.96$)	7:56:03
Failure Step 3 (Annotation)	$83.46	$101.06	$109.86	$118.66	$136.26	$153.86	**$224.26**	30 s (0.585$)	6:55:12

The cost of using spot instances with different bid prices and failure time points given for Case 2, where some spot instances get terminated (we assume half of initial number) for Exome and Whole Genome datasets. GCE cluster setup time is nearly 7 min. For every bid we provided the average lifetime of cluster in brackets. Costs in bold are the most likely ones with the respective bid price and its most likely time of failure. The best expected costs for a given experiment are underlined. The best expected cost for exome is 3.334 using bid of 0.4 and for whole genomes is 106.12 with a bid of 0.6 finishing in 33 h. If one has to finish in less than 10 h, the best price is 125.33 with 32 nodes and bid price of $0.4

The results in these table can be read as follows: If we set the bid of $0.5 for instances of type r3.8xlarge, then the expected life time is about 3 h, which is enough for running the workflow for the exome dataset without interruption. (The total time of the workflow is about 2 h as given in Table 4.). In this case the workflow would cost about $5, which is much less than that of Google (Google cost ≈ $14).

This appears fine; but in fact one can do better when using the multicloud feature of MC-GenomeKey. For example, if we reduce the bid to $0.35, then the whole workflow would cost about $2.934 assuming no failure. But there is a probability that some spot instances terminate before workflow completion, because the expected lifetime using this bid is about 100 min. In this case, the solution is to migrate the failed jobs to Google Cloud. The expected cost in case of all nodes failure would be $8.1 (Table 6) and it would be $4.8 in case of some node failure (Table 7). Both costs are less than the cost when running on Google, and in case of partial termination the cost is even less than running in a single cloud using higher bid price. Scanning all values in Table 6 in combination with the running time of each phase in the workflow given in Tables 4 and 5, one can identify that the best bid price leading to the best expected cost is $0.4 with total cost of $3.334 for all node failure and for some node failure.

Such cost advantage is more apparent when dealing with whole genome data. For example, if we take a bid price of $0.6, the workflow would cost $78 (4 nodes) - $118 (32 nodes) in case no failure of spot instances, which is much less than the $208 of Google. Even in case of failure and migration, the best prices at that bid would range between $106 (failure of some nodes in Table 7) and $149 (failure of all nodes as in Table 6). It is worth mentioning that with more nodes there is a better chance that no job fails at the first two critical phases (alignment and variant calling). I.e., the failure would occur at the easier quicker steps, which will lead to reduced time. Our estimation when using 128 nodes is that the total time would drop to about 2 h with estimated cost ranging between $103 and $130.

The data transfer times and costs are given in Tables 6 and 7. The amount of data transferred depends on the time point where the spot instances get terminated. The worst case scenario is when the sport instances get terminated at the very beginning of the workflow and in this case it is important to move most of the data from one cloud to another. For the exome data set, the worst case time is about 12 min at a cost of about $1.0. For the whole genome, the worst case data transfer time was about 230 min at a cost of about $18.0. This lead to an increase in the computation time, but the cost remained still less than using Google only. When the spot instances fail in other time points, the increase in cost and time is neglected.

To sum up, merging the spot instance model with low bid and use of Google as a host migration cloud would lead to cost reduction in many cases with minor increase in the running time.

## Conclusions

In this paper we have introduced MC-GenomeKey, a package for variant analysis and annotation using computing resources from different cloud providers.

MC-GenomeKey can run either on Google, Amazon, Azure clouds, or any combination of the three. It can also run in any cloud based on OpenStack. The package supports that the jobs of the same workflow be distributed over nodes coming from different clouds. In addition, it offers the option of handling the spot instance model by migrating jobs among the same or different clouds. The new features of the package allows easier usage through a command-line and web interface, allows faster execution through improved parallelization, and is more cost-effective via exploiting best business offers in different clouds. The associated cost reduction is a step towards the elimination of the barriers limiting the use of NGS in clinical settings.

MC-GenomeKey can be tuned to work with organisms other than human, provided that the parameters of the tools are set properly and the annotation database are put in required formats. Fortunately, the Annovar annotation system supports other organisms, such as mouse, worm, Yeast, among others. In a future version of our system, we would support other organisms of interest to the community.

It is worth mentioning that MC-GenomeKey supports that each phase of the variant analysis workflow runs in a different cloud, as specified by the configuration file (for example, mapping runs in Amazon and variant calling runs in Google). However, we did not expose this feature to the MC-GenomeKey web-interface, because it is not yet of practical relevance. The reason is that the current pricing scheme makes it always cheaper to run the whole workflow in Google if one searches for best price. Also the current machine configurations and performance make it faster to run the whole workflow in Amazon if one searches for fastest time, as shown in Table 4.

In spite of its cost advantage, the spot instance model was not considered as a useful model because of the sudden termination of nodes when the spot price exceeds the bid price. MC-GenomeKey has provided an efficient solution based on migrating the jobs associated with the terminated nodes to other on-demand nodes in the same cloud or in Google cloud. It is also worth mentioning that the robust use of spot instances can lead also to a faster execution given a certain budget ceil. That is, the reduced price allows the user to allocate more machines to finish as fast as possible within the budget limit.

Transferring the data among cloud sites is a limiting factor for some use case scenarios of multicloud. The use of high-speed data transfer solutions (either commercial like aspera or open source like GridFTP, among others) would provide an efficient solution to this problem. In the next versions of MC-GenomeKey, we will integrate high speed data transfer solutions to further improve the performance. In addition, we will also work on integrating the streaming techniques presented in [30], where the computation can take place while the data is being transferred to save more time.

While editing this manuscript Google has provided the custom instances, where the user can shape the machines specifications. Although there is no change in the price per compute unit, this will provide more flexibility in selecting the best infrastructure for certain applications. Also while working on this manuscript, Amazon introduced the concept of reserved spot instances, where the user can reserve the spot node without termination for up to 6 h with a price ranging between 50 and 100% of the equivalent on-demand price. These two examples show the severe competition between Google and Amazon and the importance of MC-GenomeKey enabling the user select best prices and options.

MC-GenomeKey is available at http://nubios.nileu.edu.eg/mcgk and https://bitbucket.org/shazly/mcgk. We are also working in integrating it in the Tavaxy workflow management system [31, 32].

## Availability and requirements 

**Project name:** MC-GenomeKey.

**Project home page:** http://nubios.nileu.edu.eg/mcgk, https://bitbucket.org/shazly/mcgk

**Operating system(s):** Linux.

**Programming language:** Python, C, C++, Java.

**Other requirements:** NA

**License:** GPL.

**Any restrictions to use by non-academics:** No restrictions.

## Abbreviations

AWS: Amazon web services
BAM: Binary alignment/mapping
BQSR: Base quality score recalibration
BWA: Burrows-wheeler aligner
CPU: Central processing unit
GATK: Genome analysis tool kit
GCE: Google compute engine
GRCh38: Genome reference consortium (human) build 38
hg19: Human genome build 19
Indel: Insertion/deletion
MAQ: Mapping and assembly with qualities
NGS: Next-generation sequencing
RAM: Random access memory
S3: Simple storage service
SNP: Single nucleotide polymorphism
VCF: Variant call format
VQSR: Variant quality score recalibration
